# The effect of acrylamide on sperm oxidative stress, total antioxidant levels, tyrosine phosphorylation, and carboxymethyl-lysine expression: A laboratory study

**DOI:** 10.18502/ijrm.v19i7.9473

**Published:** 2021-08-16

**Authors:** Mojdeh Hosseinpoor Kashani, Mina Ramezani, Zeinab Piravar

**Affiliations:** Department of Biology, Faculty of Sciences, Central Tehran Branch, Islamic Azad University, Tehran, Iran.

**Keywords:** Acrylamide, Oxidative stress, Antioxidant, Spermatozoa, Infertility.

## Abstract

**Background:**

Acrylamide (AA) is a reactive molecule produced during food processing at temperatures above 120∘C.

**Objective:**

To evaluate the impact of different concentrations of AA on human sperm parameters, oxidative stress and total antioxidant capacity (TAC).

**Materials and Methods:**

In this laboratory study, semen samples were obtained from healthy donors referred to the Taleghani Hospital, Tehran, Iran between June and July 2019. Samples were divided into four groups (n = 10/each): one control and three treatment groups (0.5, 1, and 2 mM of AA). After 2 hr of exposure to AA, the superoxide dismutase and malondialdehyde levels were measured based on colorimetric methods. The TAC was determined by the ferric-reducing antioxidant power assay. Flow cytometry was performed to measure the intracellular reactive oxygen species generation. Also, immunohistochemistry was done to determine the effect of AA on tyrosine phosphorylation and carboxymethyl-lysine expression.

**Results:**

Results of the study demonstrated that the motility and viability of spermatozoa were significantly decreased after AA exposure (p < 0.001). This decrease was also seen in the TAC and superoxide dismutase activity as well as in the phosphotyrosine percentage compared with the control (p < 0.01). However, the carboxymethyl-lysine and prooxidant activity including reactive oxygen species generation and lipid peroxidation level increased (p < 0.001).

**Conclusion:**

Overall, the results confirmed the detrimental effect of AA on human spermatozoa which may be due to oxidative stress and decreased total antioxidant levels. AA may reduce fertility by reducing sperm capacitation and motility.

## 1. Introduction

The presence of acrylamide (AA) in many commonly consumed foods such as fried and oven-cooked foods was reported in 2002 by the Swedish Authorities for the first time, which attracted considerable concern worldwide (1). AA (CH = CH-CONH2) is a white, odorless, crystalline solid which has wide application in industry as well as in wastewater treatment and soil conditioner (2). The human daily uptake of AA is estimated at 1–4 µg/kg body weight (3). The main route for the formation of AA in high carbohydrate foods such as potato chips and cereal products is the Maillard reaction (a reaction between reducing carbohydrates and free amino groups of proteins) (4).

Genotoxicity and carcinogenicity of AA have been documented especially in hormonally regulated tissues such as the mammary gland, thyroid, and testis mesothelium (5). Therefore, it is considered a global concern by the World Health Organization and has come to be known as a reproductive toxin in recent years (6, 7). Oral AA exposure induces testicular damage as well as a decrease in testosterone and epididymal sperm in rats (8). A toxicological impact was previously indicated in the male reproductive system of weaning rats when abnormal sperms and histopathological lesions appeared after AA treatment (9). Research about AA toxic effect on female mice indicated a major impact on oocyte quality, DNA methylation, reactive oxygen species (ROS) generation, and apoptosis induction (10). ROS in small amounts is essential for sperm physiological function including maturation, capacitation, and fertilization. However, when the balance between free radical activity and the body's antioxidant system is lost, oxidative stress occurs. It is suggested that the reproductive toxicity of AA is mainly due to oxidative stress (11, 12).

The sperm cell is particularly susceptible to oxidative attack due to the low volume of cytoplasm and therefore low levels of antioxidant enzymes, such as superoxide dismutase (SOD), catalase, and glutathione peroxidase. On the other hand, high levels of polyunsaturated fatty acids make this cell vulnerable to free radicals attack (13). Lipid peroxidation (LPO) caused by ROS leads to the formation of malondialdehyde (MDA) which is a stable peroxidation product in seminal plasma. MDA is a biomarker for measuring the level of oxidative stress in the cell and a well-known indicator of reduced fertility and sperm dysfunction (14).

Oxidative stress impairs sperm motility, the sperm membrane, and DNA integrity (15, 16). AA is known to cause oxidative stress in the human body and numerous studies have indicated its toxic effect on the male reproductive system particularly in mice and rats (8, 11). But the impact of AA on human spermatozoa ROS generation total antioxidant levels and sperm motility and capacity is not fully understood yet.

Tyrosine phosphorylation has been used as a hallmark to determine the influence of this substance on sperm capacitation (17). Also, it is considered that carboxymethylation of lysine residues in the sperm tail is involved in sperm movement regulation because of its crucial role in post-translational modification (15, 18).

This study highlights the effect of different concentrations of AA on ROS generation, LPO, total antioxidant capacity (TAC) levels, and human spermatozoa parameters. The rate of LPO was measured by MDA levels. In addition, phosphorylation of tyrosine as a capacitation indicator and carboxymethyl-lysine expression were evaluated.

## 2. Materials and Methods

### Sperm preparation

In this laboratory study, human semen samples (n = 40) were collected from fertile men referred to the Taleghani Hospital, Tehran, Iran, by masturbation after at least 48 hr of abstinence. “Fertile” was defined as having a formerly pregnant partner with a known time of how long it took to get pregnant of up to 12 months. The exclusion criteria were smoking and having infertility problems. Samples were transferred to the laboratory within 1 hr of ejaculation and purification was performed by Percoll discontinuous gradient. Semen samples were placed on the topmost layer of the 40% and 80% Percoll gradient (semen: 40% Percoll: 80% Percoll = 1:3:3, v/v) and centrifuged at 400× g for 15 min.

The cell pellet was washed with a 10% fetal bovine serum (FBS, Merck, Germany) culture medium and recentrifuged. Then, samples were divided into four groups including a control and three treatment (T1–T3) groups (0.5, 1, and 2 mM AA, Merck KGaA, Germany diluted in the DMEM/F12, (Merk, Germany) medium, respectively). Next, 200 μl of the sperm solution was added to 200 μl of AA solution and incubated for 2 hr at 37∘C (15).

### Sperm motility, viability, and morphology

Sperm motility was assessed using a phase contrast microscope (Nikon, Japan) by manual observation of 100 cells at ×250 magnification. Motility was classified into one of the three movements – progressive (grades A and B), nonprogressive (grade C), and immotile (grade D) sperms. The sperm parameters were analyzed according to the World Health Organization criteria (19).

The vitality of spermatozoa was evaluated by using the vital stain Trypan blue, in accordance with previous study (20). 10 λ Trypan blue was mixed with 10 λ supernatant of sperm and 2 λ formalin 10% diluted. Trypan blue penetrates the postacrosomal region of dead cells. A drop of stained samples was placed on the slides and examined under ×1000 magnification. In each group, 200 sperm heads were counted, and the percentage of viable spermatozoa (unstained) was calculated.

### MDA assay

Seminal MDA levels were determined using the Zell Bio colorimetrical kit (GmbH, Germany) according to the manufacturer's protocol. The kit's sensitivity was equal to 0.1 mM. It uses the reaction of MDA and thiobarbituric acid under a high temperature. MDA was measured in an acidic media and heat (90–100∘C) colorimetric ally at 535 nm.

### SOD activity assay

Seminal SOD activity levels were determined using the Zell Bio colorimetrical kit (GmbH, Germany) according to the manufacturer's protocol. The kit's sensitivity was equal to 0.044 U/mL. It uses a xanthine oxide reagent at room temperature. This substance generates superoxide in the presence of oxygen that converts a colorless substrate to a yellow color product. By increasing the levels of SOD in the sample, a decrease in superoxide concentration and yellow color occurs. Then, samples are read at 450 nm wavelength.

### TAC assay

To measure the TAC, the ferric-reducing antioxidant power (FRAP) assay kit (Cell Biolabs, USA) was used according to the manufacturer's instructions. This method can detect antioxidant capacity as low as 0.2 mM Fe${}^{2+}$ equivalent using colorimetry. The basis of the assay is the reduction of ferric iron (Fe${}^{3+}$) to ferrous iron (Fe${}^{2+}$) by antioxidants present in the sample. At the end of the experiment, the probe of the kit developed a blue color that was read at 540–600 nm in this case. The antioxidant potential of samples was determined based on an iron standard curve and results were calculated at Fe${}^{2+}$ equivalents (µM) or FRAP value.

### ROS assay

The dichlorofluorescin diacetate flow cytometry method was performed to measure the intracellular ROS generation after exposure of the sperm samples to AA, according to the manufacturer's instructions (Abcam, USA).

Dichloro-dihydro-flourescein diacetate DCFH-DA chemically reacts with ROS and produces a highly fluorescent compound called dichlorofluorescein which exits the cell. Finally, the fluorescent intensity was measured by FACSDiva (BD Bioscience, USA) flows cytometry. Data were analyzed using Flowjo software version 10.7 (BD Bioscience, USA).

### Immunohistochemistry

#### Carboxymethyl-lysine

After treatment with AA, sperm samples (5 × 10${}^{6}$/ml) were fixed with 4% paraformaldehyde. The samples were air-dried and washed 4× in phosphate buffered saline (PBS, Merck, Germany). To recover the antigen, 2 N hydrochloric acid (Merck, Germany) was poured on the samples for 30 min. Then, 1 mM borate buffer (Merck, Germany) was added to neutralize the acid for 5 min and the cells were washed with PBS.

The samples were permeabilized with 0.3% Triton for 30 min. The blocking agent was 10% goat serum for 30 min at room temperature. Primary antibody (mouse monoclonal anticarboxymethyl-lysine antibody, ab125145, USA) was diluted (1/100) in bovine serum albumin (Merck, Germany) overnight and incubated in a fridge in a humidified chamber. Samples were washed 4× in PBS and incubated with secondary goat anti-mouse IgG (Alexa Flour 488, abc150113) at dilution 1/150 in Bovine Serum Albumin (Merck, Germany) at 37∘C for 1 hr and 30 min in a dark place. Further, the slides were washed 3× in PBS and exposed to DAPI (1/2000) for 10 min at room temperature, then washed with PBS. Sperms were examined under a fluorescence microscope (Olympus, Japan) using a ×400 objective. On each study slide, 200 sperm cells were evaluated for confirmation of markers (antibody expression and nucleus staining).

#### Phosphotyrosine

After treatment with AA, sperm samples (5 × 10${}^{6}$/ml) were capacitated with dcAmp and pentoxifylline at 37∘C for 3 hr. Then the samples were treated as explained earlier. The only difference was in the primary and secondary antibodies which were P-T-YR and goat anti-rabbit IgG (abc150077), respectively.

### Ethical considerations

The scientific use of the samples was approved by the Research Ethics Committee of Medical Science (Code: IR.IAU.TMU.REC.1398.091). Semen samples were collected from healthy men with at least one child. Written informed consent was obtained from all subjects.

### Statistical analysis

All experiments were done with three replications. Data were analyzed by Graphpad Prism version 8.4.3 (686) (Graphpad, USA) using one-way ANOVA, followed by Tukey-Kramer post hoc tests. The difference in the mean values at p < 0.05 was considered as significant.

## 3. Results

### Impact of AA on human spermatozoa parameters

Exposure of human spermatozoa to AA caused a significant decrease in the percentage of progressive spermatozoa and the total motility at all doses of AA. Also, the percentage of immotile spermatozoa was significantly increased at high doses in comparison with the control (Tables I and II). In addition, the viability of the spermatozoa decreased significantly compared with the control.

### Impact of AA on MDA, TAC, and SOD levels

Table III demonstrates the significant increase in the LPO and MAD production observed at all doses of AA (p < 0.01). Conversely, the levels of TAC and SOD activity showed a significant decrease especially at high doses of AA.

### Impact of AA on ROS levels

**Figure 1:**
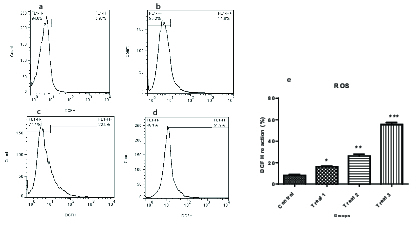
Dichlorofluorescin diacetate flow cytometry in human spermatozoa after exposure to different concentrations of AA (a: 0, b: 0.5, c: 1, and d: 2 mM). Comparison of human spermatozoa ROS levels in different groups of treatment (e).

Results of flow cytometry revealed that after exposure of spermatozoa to different concentrations of AA, the ROS levels increased significantly (Figure 1, and Table IV).

### Impact of AA on carboxymethyl-lysine expression

**Figure 2:**
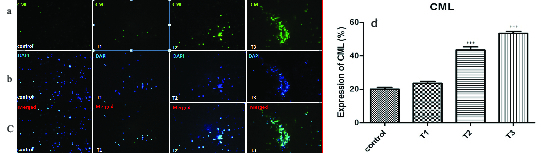
Immunofluorescent staining of carboxymethyl-lysine expression in the control and AA-treated groups (T1–T3). (a) Expression of carboxymethyl-lysine antibody can be seen with a green color by the fluorescent microscope. (b) DAPI was added to examine the presence of sperm cells. The sperm nuclei were stained by blue color. (c) Combination of both protein expression and nucleus staining. (d) Comparison of carboxymethyl-lysine expression in different treatment groups.

Figure 2 and Table V show the expression of carboxymethyl-lysine in the tail of the human spermatozoa in the control and acrylic-treated groups (T1–T3). By increasing the concentration of AA, the percentage of protein expression increased: at 1 and 2 mM concentrations, the expression of carboxymethyl-lysine was significantly higher than in the control.

### Impact of AA on phosphotyrosine expression

**Figure 3:**
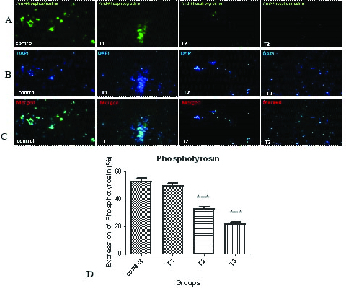
Immunofluorescent staining of phosphotyrosine in the control and AA-treated groups (T1–T3). (a) Expression of phosphotyrosine antibody can be seen with a green color by the fluorescent microscope. (b) DAPI was added to examine the presence of sperm cells. The sperm nuclei were stained by blue color. (c) Combination of both protein expression and nucleus staining. (d) Comparison of the percentage of phosphotyrosine expression in different treatment groups.

Figure 3 and Table VI show protein phosphorylation in tyrosine amino acid residues was indicated in the control and AA-treated groups (T1–T3) using immunohistochemistry. By increasing the concentration of AA, the percentage of tyrosine phosphorylation decreased significantly at concentrations of 1 and 2 mM of the AA.

**Table 1:** Analysis of the sperm parameters after 2 hr of exposure with different concentrations of AA (0.5, 1, and 2 mM)


**Variables (%)**	**Control**	**Treatment 1**	**Treatment 2**	**Treatment 3**
**Progressive motility**	73.81 ± 1.18	62.63 ± 2***	44.61 ± 0.6***	34.62 ± 0.7***
**Total motility**	87.50 ± 1.5	82.70 ± 2.25	72.23 ± 1.96***	68.72 ± 1.9***
**Immotile**	12.5 ± 1.5	16.77 ± 1.2	27.29 ± 1.25	31.28 ± 0.95**
**Viability**	95.98 ± 0.52	84.09 ± 1.7***	76.47 ± 1.16***	64.06 ± 1.33***
Data are presented as Mean ± SEM and analyzed by Tukey-Kramer test. **P < 0.01, ***P < 0.001				

**Table 2:** The difference of sperm parameters in AA treatment and control groups


**–**		**Mean difference**	**p-value**	**95% CI of the difference**
**Progressive motility**				
	**Control vs. treatment 1**	11.18	0.001	6.346–16.02
	**Control vs. treatment 2**	29.21	0.001	24.37–34.04
	**Control vs. treatment 3**	39.20	0.001	34.36–44.04
**Total motility**				
	**Control vs. treatment 1**	4.797	–	–2.528–12.12
	**Control vs. treatment 2**	21.27	0.001	13.94–28.59
	**Control vs. treatment 3**	34.78	0.001	27.46–42.11
**Immotile**				
	**Control vs. treatment 1**	–1.269	–	–7.199–4.661
	**Control vs. treatment 2**	–4.795	–	–10.72–1.135
	**Control vs. treatment 3**	–8.785	0.01	–14.71–2.855
**Viability**				
	**Control vs. treatment 1**	11.88	0.001	7.107–16.66
	**Control vs. treatment 2**	19.50	0.001	14.73–24.28
	**Control vs. treatment 3**	31.91	0.001	27.14–36.69
Tukey's multiple comparison test				

**Table 3:** Comparison of MDA, TAC, and SOD concentrations in the control and AA (0.5, 1, and 2 mM) exposed groups


**Variables **	**Control**	**Treatment 1**	**Treatment 2**	**Treatment 3**
**MDA (nmol/ml)**	17.55 ± 2.5	33.26 ± 5.6**	44.33 ± 4.3**	59.69 ± 3.4***
**TAC (µM/l)**	0.434 ± 0.06	0.320 ± 0.08	0.213 ± 0.08*	0.165 ± 0.04**
**SOD (IU/ml)**	27.62 ± 2.67	20.15 ± 2.56**	13.99 ± 1.32**	6.96 ± 3.86**
Data are presented as Mean ± SD and analyzed by Tukey-Kramer test. *P < 0.05, **P < 0.01, ***P < 0.001. MDA: Malondialdehyde, TAC: Total antioxidant capacity, SOD: Superoxide dismutase				

**Table 4:** The difference of ROS levels in AA treatment and control groups


**ROS**	**Mean difference**	**q-value**	**p-value**	**95% CI of the diff**
**Control vs. treatment 1**	11.18	8.813	0.05	6.346–16.02
**Control vs. treatment 2**	29.21	23.01	0.01	24.37–34.04
**Control vs. treatment 3**	39.20	30.89	0.001	34.36–44.04
Data analyzed by Tukey's Multiple Comparison Test. ROS: Reactive oxygen species				

**Table 5:** The difference of carboxymethyl-lysine expression in AA treatment and control groups


**CML**	**Mean difference**	**q-value**	**p-value**	**95% CI of the difference**
**Control vs. treatment 1**	-3.430	2.459	- –9.28–2.427
**Control vs. treatment 2**	-23.38	16.76	0.001	–29.24–17.52
**Control vs. treatment 3**	-33.45	23.98	0.001	–39.30–27.59
Data analyzed by Tukey's Multiple Comparison Test. CML: Carboxymethyl-lysine				

**Table 6:** The difference of phosphotyrosine in AA treatment and control groups


**Phosphotyrosine**	**Mean difference**	**q-value**	**p-value**	**95% CI of the difference**
**Control vs. treatment 1**	3.095	1.986	–	–3.450–9.640
**Control vs. treatment 2**	20.16	12.93	0.001	13.61–26.70
**Control vs. treatment 3**	31.05	19.92	0.001	24.50–37.59
Tukey's Multiple Comparison Test				

## 4. Discussion

So far, studies on the effect of AA on fertility are limited to rodent models (21, 22). The present research is the first comprehensive study on the effect of AA on human spermatozoa. There were no major limitations in this study. According to the findings of this study, all doses of AA significantly reduced sperm viability and motility. The results of the present work are consistent with the results of a previous study about the effect of oral treatment of AA on the mouse spermatozoa. It seems that AA, through its effects on membrane integrity, decreases sperm vitality, causing sperm movement disorder such as abnormal sperm parameters in total and progressive motility (21).

Alterations in the cellular redox status may occur through an imbalance between oxidant and antioxidant systems in the semen; therefore, we investigated the rate of oxidative impairment both with the positive factor of antioxidant enzyme activity and the negative factor of oxidative stress correlated with the sperm parameters.

It has been reported that the coexistent of AA and trans fatty acids (which exist in many thermally processed foods) enhances the oxidative stress in mice epididymal sperm by upregulating the MDA levels and reducing the SOD activity, as well as glutathione peroxidase (23).

In our previous study, it was found that the mitochondrial membrane potential decreased after 2 hr of sperm incubation with AA (24). On the other hand, AA could inhibit the activity of glotation s-transferase enzymes (a family of phase-II detoxification enzymes that catalyze the conjugation of glutathione) leading to cell membrane damage. These changes also appear to be due to an increase in the cellular ROS content which disrupts the process of oxidative phosphorylation in the mitochondria and the production of ATP in the cells (24, 25). As a result, it is associated with reduced motility, inhibition of the acrosomal reaction, and reduced sperm ability for fertilization (26).

AA exerts its effects at least in some parts by weakening the antioxidant status in the sperm cells (22). It was reported that exposure to AA in mice caused reduced sperm motility and impaired fertility (27). As has been mentioned, LPO is one of the major mechanisms of cellular damage caused by ROS, as the sperm membrane is rich in unsaturated fatty acids that are very sensitive to free radicals. MDA is the main indicator of LPO identification (14). Our data also confirmed an increase in MDA levels following AA exposure.

The proposed mechanism for the effect of ROS on sperm function and structure is through inhibition of the activity of enzymes such as glucose 6 phosphate dehydrogenase (G6PD). The produced H${}_{2}$O${}_{2}$ passes through the membrane, enters the cell, and inhibits the activity of this enzyme. G6PD controls the influx of glucose through the pentose phosphate pathway which in turn controls the intracellular presence of NADPH. NADPH acts as an electron source for the spermatozoa to produce ROS by the enzyme known as NADPH oxidase (28). The inhibition of G6PD reduces NADPH and causes simultaneous accumulation of glutathione oxidase and reduction of glutathione. This can enhance phospholipid peroxidation in the sperm which decreases membrane fluidity and integrity (29). As a result of this, the antioxidant defense system is disrupted which can lead to defective sperm function.

Carboxymethylation of spermatozoa tail proteins on lysine residues is a post-translational modification which is necessary for normal sperm development and movement. Carboxymethyl-lysine is a common and potent advanced glycation end (AGE) compound on all parts of sperm cells particularly in the head region that is surrounded by the acrosomal region (18). Increased levels of the AGEs lead to sperm damage mostly through defects in DNA integrity (15). In this study, immunohistochemistry of carboxymethyl-lysine demonstrated a significant increase after 2 hr of exposure with 1 and 2 mM of AA. An increase in the AGEs compounds which is produced during the Maillard reaction is detrimental to health and fertility (30). Therefore, diet seems to be one of the major factors reducing semen quality and male fertility. As mentioned earlier, fast foods and thermally processed foods are the major sources of AA production. One of the aggravating factors for AGE formation is oxidative stress (31). In the present research, AA produced oxidative stress by increasing the ROS and MDA levels and decreasing the TAC and SOD. This means that AA probably exerts its effects by increasing carboxymethyl-lysine expression and raising oxidative stress in the sperm cell.

Capacitation is a complex event in the female reproductive system during which the sperms acquire fertilization potential. It has been reported that modifications including an increase in tyrosine phosphorylation of sperm membrane proteins are necessary for capacitation. The pattern of localization of phosphorylated tyrosine has been shown in the neck and flagellum of the sperm by immunofluorescence studies (15). Tyrosine phosphorylated sperm proteins in the flagellum are related to hyperactivated motility which is initiated during in vitro capacitation (17). As a result, abnormalities in sperm motility due to decreased tyrosine phosphorylation levels are not uncommon. The immunohistochemistry results of the present study also showed a decrease in the phosphotyrosine levels.

## 5. Conclusion

The results of this study indicated that different concentrations of AA had a negative effect on the motility and viability of human spermatozoa. AA could reduce the sperm motility and ability for capacitation by increasing the ROS levels relative to the cell's antioxidant capacity. Therefore, given the rising male infertility rate and increasing consumption of deep-fried foods containing AA, more attention should be paid to the effects of AA on male infertility.

##  Conflicts of Interest

There is no conflict of interest.
